# Staurosporine, an inhibitor of hormonally up-regulated neu-associated kinase

**DOI:** 10.18632/oncotarget.26311

**Published:** 2018-11-13

**Authors:** Joelle N. Zambrano, Christina J. Williams, Carly Bess Williams, Lonzie Hedgepeth, Pieter Burger, Tinslee Dilday, Scott T. Eblen, Kent Armeson, Elizabeth G. Hill, Elizabeth S. Yeh

**Affiliations:** ^1^ Department of Cell and Molecular Pharmacology and Experimental Therapeutics, Medical University of South Carolina, Charleston, SC 29425, USA; ^2^ Department of Drug Discovery and Biomedical Sciences, Medical University of South Carolina, Charleston, SC 29425, USA; ^3^ Department of Chemistry, Emory University, Atlanta, GA 30322, USA; ^4^ Department of Public Health Sciences, Medical University of South Carolina, Charleston, SC 29425, USA

**Keywords:** HUNK, staurosporine, HER2, breast cancer, resistance

## Abstract

HUNK is a protein kinase that is implicated in HER2-positive (HER2+) breast cancer progression and resistance to HER2 inhibitors. Though prior studies suggest there is therapeutic potential for targeting HUNK in HER2+ breast cancer, pharmacological agents that target HUNK are yet to be identified. A recent study showed that the broad-spectrum kinase inhibitor staurosporine binds to the HUNK catalytic domain, but the effect of staurosporine on HUNK enzymatic activity was not tested. We now show that staurosporine inhibits the kinase activity of a full length HUNK protein. Our findings further suggest that inhibiting HUNK with staurosporine has a strong effect on suppressing cell viability of HER2/neu mammary and breast cancer cells, which express high levels of HUNK protein and are dependent on HUNK for survival. Significantly, we use *in vitro* and *in vivo* methods to show that staurosporine synergizes with the HER2 inhibitor lapatinib to restore sensitivity toward HER2 inhibition in a HER2 inhibitor resistant breast cancer model. Collectively, these studies indicate that pharmacological inhibition of HUNK kinase activity has therapeutic potential for HER2+ breast cancers, including HER2+ breast cancers that have developed drug resistance.

## INTRODUCTION

Human Epidermal Growth Factor Receptor 2-positive (HER2+) breast cancer accounts for 15–30% of all breast cancer diagnoses. This subtype is associated with poor prognosis and it is reported that there is a high incidence of resistance to clinically-available HER2 inhibitors [1–5]. Hormonally Up-regulated Neu-associated Kinase (HUNK) is a Serine (Ser)/Threonine (Thr) kinase that is highly expressed in HER2+ breast cancers and HER2/neu+ transformed breast or mammary tumor cell lines [6–8]. HUNK has been shown to promote survival in HER2+ breast cancer cells [6, 9, 10], and to promote mammary tumorigenesis *in vivo* [6, 9–11]. Recent reports have also shown that using shRNA to impair HUNK expression suppresses *in vivo* tumor growth of orthotopic mammary tumors generated from HER2+ breast cancer cells that are resistant to HER2 inhibitors [9, 10]. These studies suggest that targeting HUNK could be advantageous in the treatment of HER2+ breast cancer, particularly in cases where HER2 inhibitor resistance is indicated.

Because no pharmacological inhibitors that target HUNK kinase activity have been identified, prior studies that look at the kinase activity of HUNK make use of kinase-inactive mutants of HUNK; for example, a HUNK protein containing a point mutation at lysine 91 (K91M HUNK) [6, 8, 11]. Previous studies have shown that expression of the K91M kinase-dead mutant of HUNK significantly impairs tumor growth in an MMTV-neu model [6]. These studies indicate HUNK kinase activity is essential for promoting HER2/neu mammary tumor growth, and suggest that pharmacological inhibition of HUNK kinase activity has potential as a therapeutic option for this subtype of breast cancer.

In a 2011 *Nature Biotechnology* publication, Ambit Biosciences published a study on their KINOMEscan™ competition binding assay platform (now owned by DiscoverRx), which evaluated 72 inhibitors against 442 kinases [12]. Using the isolated kinase domain of HUNK, this study identified 10 compounds, which bound to the HUNK kinase domain with high binding affinity (≤1500 nM K_d_). However, these studies did not evaluate if binding of these compounds to the HUNK kinase domain inhibited enzymatic activity. Based on these findings, we now provide evidence to show that the compound, staurosporine (STU), inhibits HUNK kinase activity. Our findings show that STU reduces cell viability of HER2/neu+ breast and mammary tumor cells as well as HER2-inhibitor (trastuzumab and lapatinib) resistant HER2+ human breast cancer cells, which are dependent on HUNK as a pro-survival signaling molecule. Additionally, we report that STU exhibits synergistic cell killing effects with the dual EGFR/HER2 inhibitor lapatinib on HER2-inhibitor resistant breast cancer cells. We further confirmed our *in vitro* findings by demonstrating that mice receiving low dose combination treatment of STU and lapatinib displayed reduced mammary tumor growth of HER2+ resistant breast cancer cells compared to either inhibitor alone. Taken together, our results indicate HUNK inhibition using a pharmacological inhibitor may be a novel therapeutic approach for HER2+ breast cancer and that targeting HUNK in conjunction with HER2 inhibition will be beneficial for the treatment of refractory HER2+ breast cancer.

## RESULTS

### Staurosporine inhibits HUNK kinase activity

**Figure 1:**
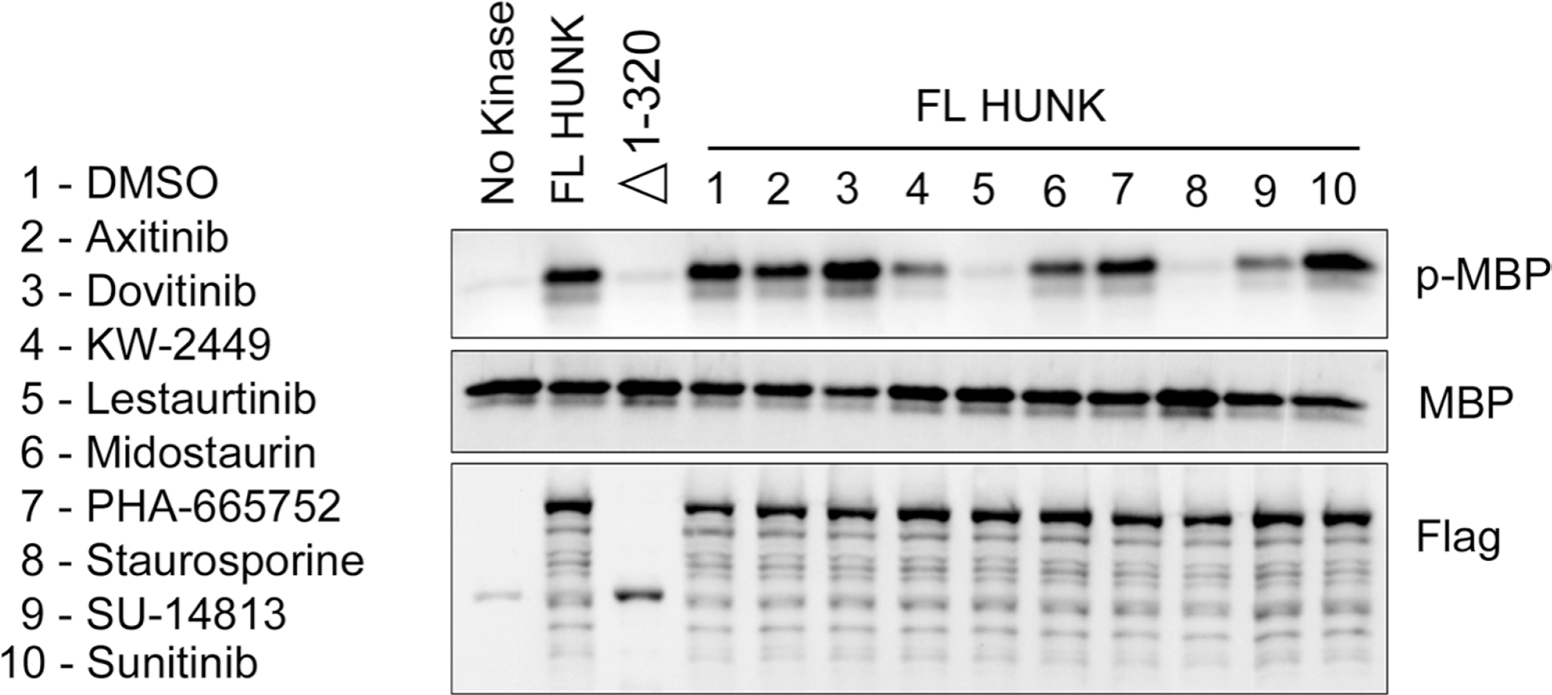
HUNK kinase assay testing the activity of compounds that bind to HUNK kinase domain Flag-HUNK was transfected into 293T cells and isolated using Flag-M2 beads. No kinase control was a mock IP using Flag-M2 beads incubated with untransfected lysate. Flag-321-ST HUNK lacks the kinase domain. Immunoblot to detect phosphorylation of MBP by HUNK was performed using a phospho-MBP (p-MBP) antibody. Flag-HUNK was pre-treated with either DMSO (vehicle) or 5 µM of compound as indicated prior to the addition of MBP.

A prior study from Davis *et al.* showed that 10 compounds (Table 1) from a group of 72 total inhibitors tested, bind HUNK’s catalytic domain with high affinity [12]. However, these studies used an isolated fragment of HUNK containing only the kinase domain and did not evaluate if binding of these compounds to the HUNK kinase domain inhibited enzymatic activity of HUNK. Therefore, we sought to determine if any of the 10 compounds found to bind the isolated HUNK kinase domain inhibit the kinase activity of the full length protein. We evaluated the effect of the 10 selected compounds on HUNK kinase activity by performing an *in vitro* kinase assay using a Flag-tagged full length HUNK that was expressed and immunoprecipitated from 293T cells. As negative controls, we used a deletion mutant of HUNK that does not contain the kinase domain (Δ 1–320) as well as a no kinase control sample. Isolated Flag-HUNK was incubated with either DMSO (vehicle) or each compound as indicated in the presence of ATP, prior to initiating the kinase reaction. Myelin basic protein (MBP) served as the substrate. Using a phospho-specific antibody to MBP to detect phosphorylated MBP, we found that 4 of the 10 compounds had some level of inhibition toward HUNK kinase activity (Figure 1), with 2 of these compounds having a modest effect on kinase activity (KW-2449 and SU-14813) and 2 of these compounds having a significant effect on kinase activity (lestaurtinib and staurosporine).

**Table 1:** Compounds with high affinity binding for the isolated HUNK kinase domain

Compound	Affinity K_d_ (nM)	Targets
SU-14813	3.7	VEGFR, FLT1, PDGFRB
Midostaurin	240	PKCα/β/γ, SYK, FLK-1, AKT, PKA, FLT3, VEGFR1/2
NVP-TAE684	350	ALK
Dovitinib	410	FLT3, FGFR3, VEGFR1-4
KW-2449	460	FLT3, ABL1 (T315I), AURKA
Sunitinib	500	KIT, VEGFR2, FLT3
Lestaurtinib	570	FLT3, TRKA/B/C, JAK2
Staurosporine	620	PRKCH (Pan-PKC inhibitor)
PHA-665752	1400	MET
Axitinib	1500	VEGFR1-3, PDGFRA/B, KIT

**Figure 2:**
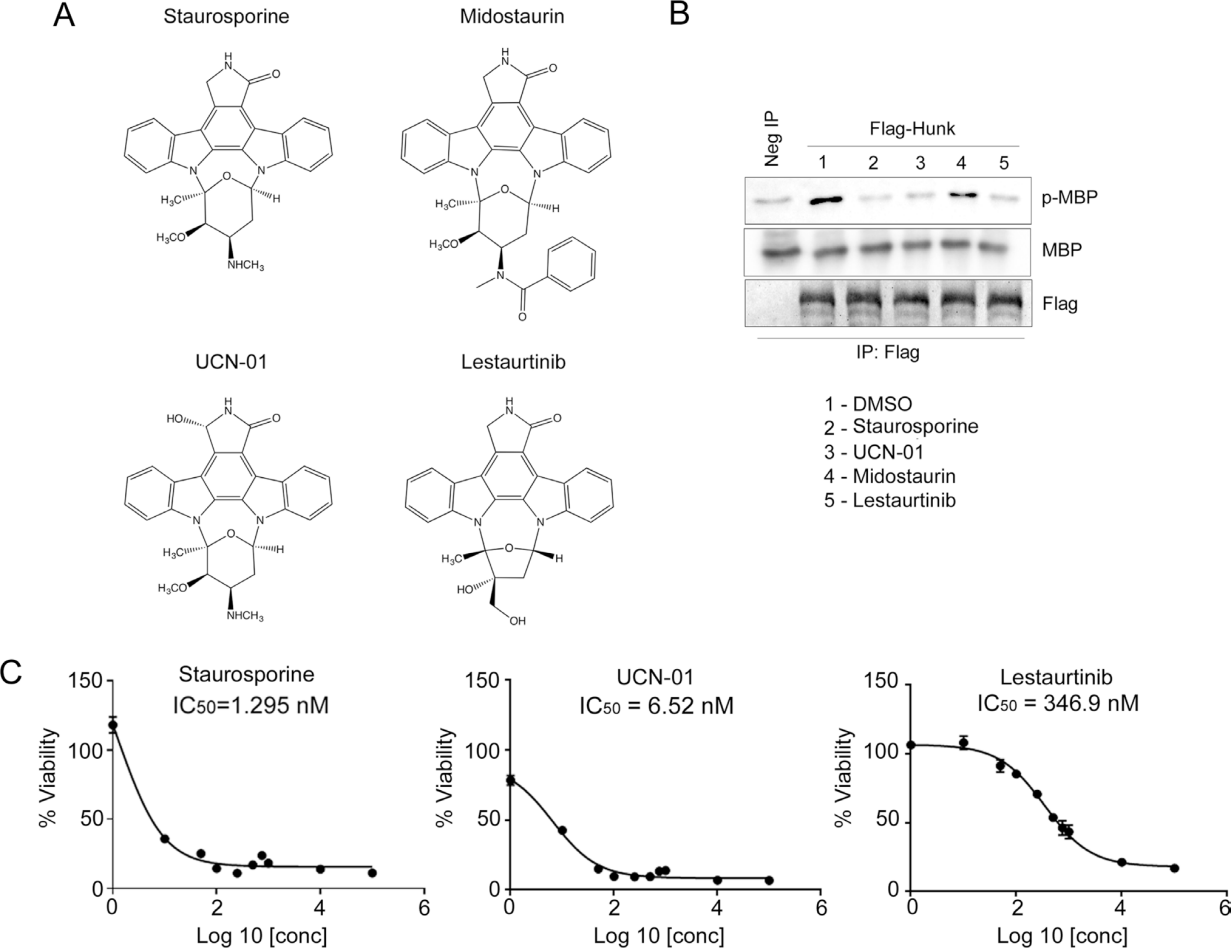
Staurosporine inhibits HUNK kinase activity (**A**) Chemical structures of indolocarbazole compounds, staurosporine (STU), midostaurin, UCN-01, and lestaurtinib. (**B**) Flag-HUNK was transfected into 293T cells and isolated using Flag-M2 beads. No kinase control was a mock IP using Flag-M2 beads incubated with untransfected lysate. Immunoblot to detect phosphorylation of MBP by HUNK was performed using a phospho-MBP (p-MBP) antibody. Flag-HUNK was pre-treated with either DMSO (vehicle) or 1 µM of compound as indicated prior to the addition of MBP. (**C**) IC_50_ values of staurosporine, UCN-01 and lestaurtinib were generated by evaluating viability of cells treated with increasing doses of compound.

Interestingly, stauroporine (STU) and lestaurtinib are structurally related compounds. Figure 2A shows the chemical structure of STU and three related analogs, lestaurtinib and midostaurin, which were identified in the Davis *et al.* study and 7-hydroxystaurosporine (a.k.a. UCN-01), which was not included in the Davis *et al.* study [12]. Consistent with Figure 1, STU, UCN-01, and lestaurtinib prevented HUNK from phosphorylating MBP, while midostaurin did not (Figure 2B).

### Staurosporine impairs cell viability of MMTV-neu-derived mammary tumor cells

Prior studies show that HUNK is overexpressed in HER2/neu+ breast or mammary tumor cell lines and that HUNK-deficient breast and mammary tumor cells have reduced viability [6, 9–11]. Therefore, we next tested each of the inhibitors that showed HUNK inhibition; STU, UCN-01, and lestaurtinib, for their ability to suppress cell viability. SMF (MMTV-neu) murine derived mammary tumor cells, which have high HUNK expression levels [6], were treated with increasing doses of STU, UCN-01, or lestaurtinib. STU and UCN-01 showed strong anti-proliferative effects, with IC_50_ values in the low nanomolar (nM) range (∼1.5 nM and ∼6.5 nM respectively), compared to lestaurtinib (Figure 2C), which had a higher (∼350 nM) calculated IC_50_ value. Though each of the compounds negatively affected viability, STU’s effects were more robust compared to UCN-01 and lestaurtinib. Because STU showed the strongest effects on cell viability, we focused on this inhibitor for the remainder of our experiments.

### Receptor-based modeling of the interaction between staurosporine and the HUNK kinase domain

**Figure 3:**
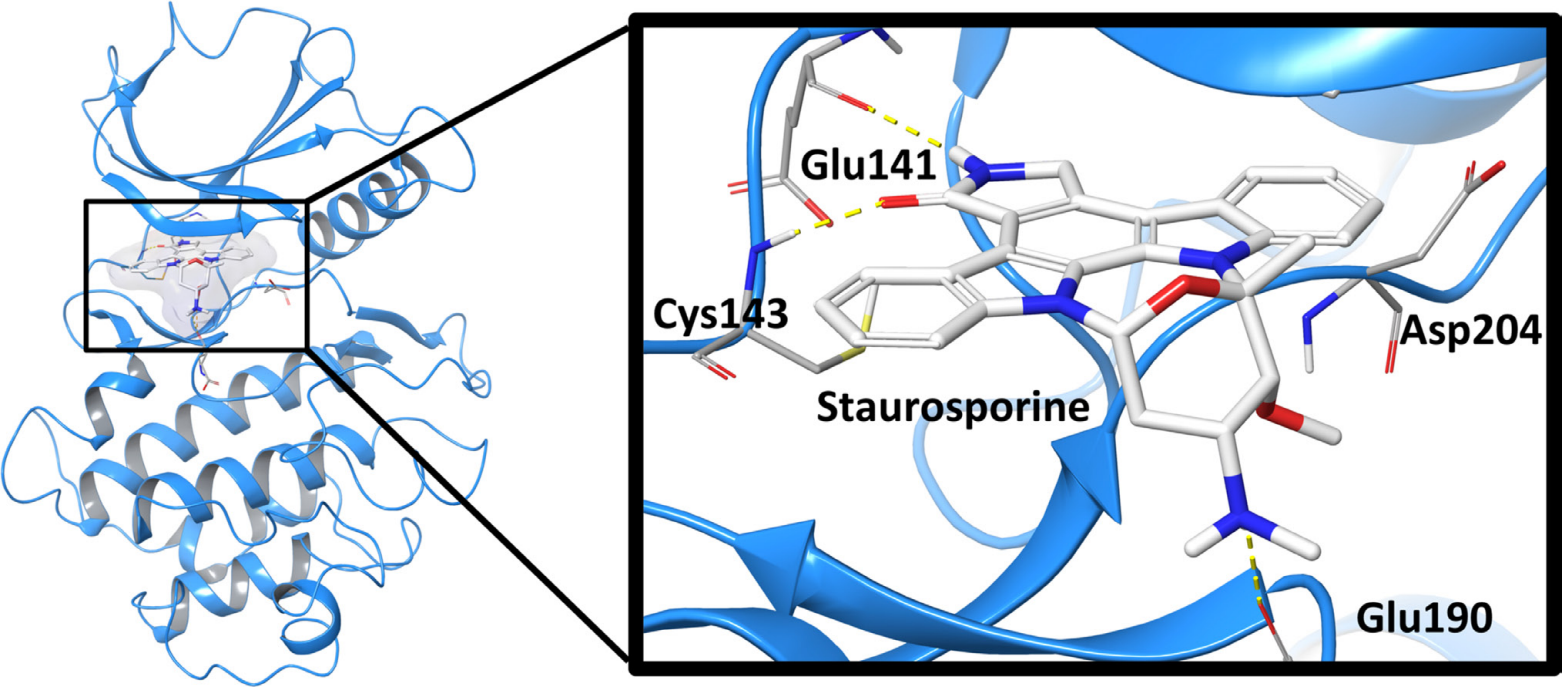
Computational model of STU bound to HUNK A modeled representation of the ATP binding pocket of the human HUNK kinase (blue) bound with STU (white). Hydrogen bonds formed between HUNK and STU in yellow.

Due to a lack of a crystal structure for HUNK, we turned to structure-based computational aided drug design (CADD) methods to evaluate the interaction between STU and the human HUNK kinase domain. STU, lestaurtinib, UCN-01 and midostaurin belong to the indolocarbazole class of compounds. Homology models representative of the interactions between the human HUNK kinase domain and the indolocarbazole compounds were generated from two serine/threonine kinases template structures co-resolved with STU. The first, being the human Checkpoint kinase 1 (Chk1; PDB entry 1NVR) and the second, being the human Death association protein kinase 1 (DAPK1; PDB entry 1WVY). HUNK shares a 31.3% and 55.5% sequence identity and a 32.3% and 54.8% sequence similarity with the kinase domain of Chk1 and DAPK1, respectively. The Chk1 structure was selected since it was co-resolved with STU at high resolution (1.8 Å) whereas the DAPK1 (2.8 Å) structure was selected because it shared a common G-rich motive in the ATP-binding loop of HUNK. Protein quality assessment showed that the model had a G-factor value of –0.33 (values ≤ –0.5 are unusual), suggesting that our model is of reasonable quality. A short molecular dynamics run at 100 K was performed to alleviate molecular constraints and equilibrate the HUNK structure containing STU prior to docking studies. The four indolocarbazole derivatives were docked to the HUNK homology model and the interactions with STU are shown in Figure 3. Similar binding poses were obtained for three of the indolocarbazole compounds as expected from their rigid nature and relatively few strong electrostatic features. Midostaurin did not return a docking pose and this finding is in agreement with our experimental results showing that midostaurin does not have activity against HUNK.

### Staurosporine treatment of high HUNK expressing MMTV-neu mammary tumor cells results in the same functional outcome as HUNK inhibition in these cells

**Figure 4:**
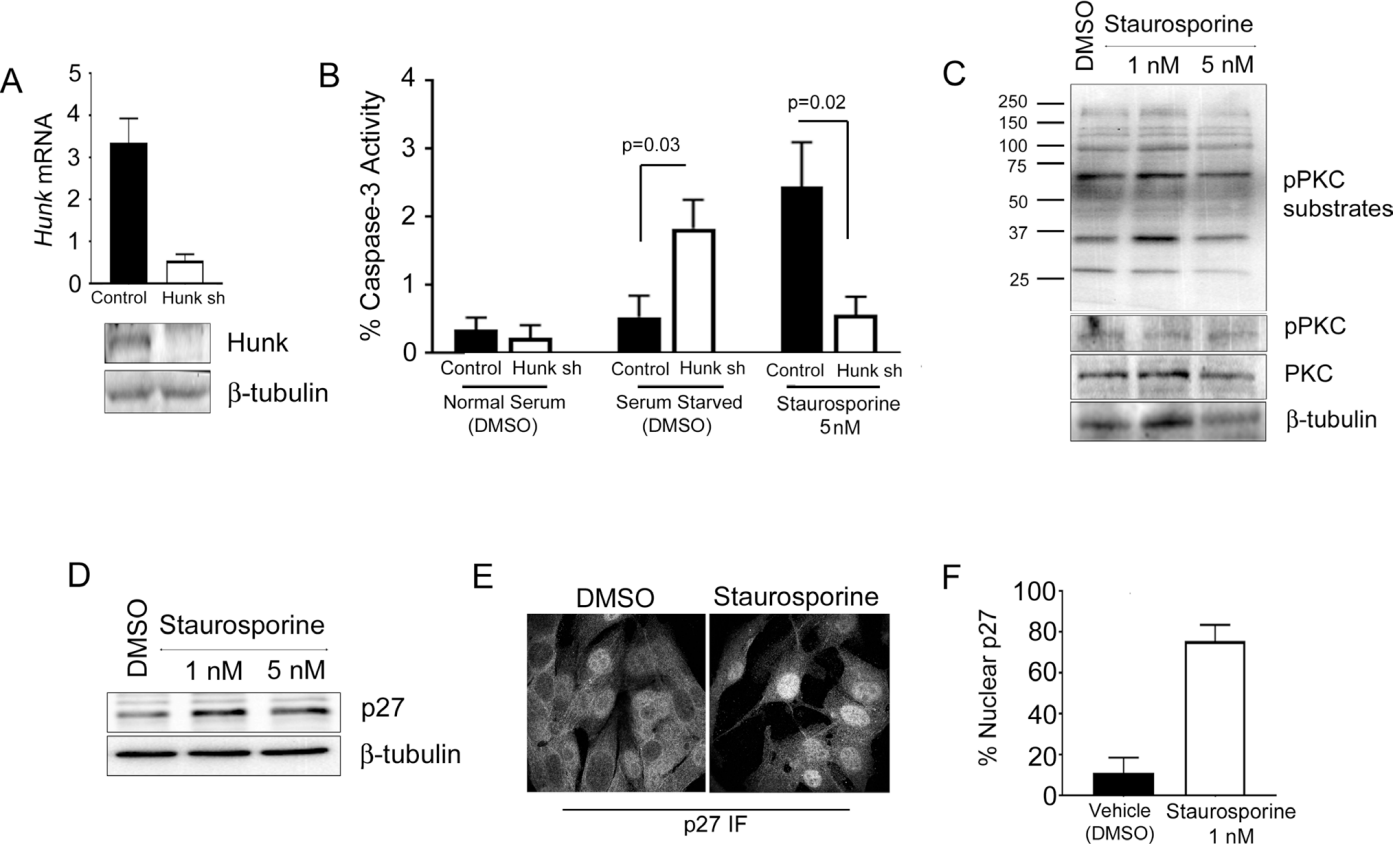
STU has similar activity in cells as HUNK inhibition (**A**) Control and HUNK knockdown SMF cells expressing a control shRNA or a shRNA targeting mouse HUNK (*Hunk)* were evaluated for *Hunk* mRNA expression levels by Quantitative-RealTime PCR to show the efficiency of knockdown. *Hunk* levels were normalized to *Gapdh.* Western blot analysis of HUNK showed reduced protein expression in *Hunk* shRNA cells compared to control cells. (**B**) Control and HUNK knockdown SMF cells were treated with either DMSO, growth factor deprivation (0% FBS, no insulin), or STU for 24 hours and analyzed for caspase-3 activity (**C**) SMF cells were treated with either DMSO or STU for 24 hours at the indicated concentration. Immunoblotting for PKC activity was performed using anti-pPKC substrate and pPKC antibodies. Total PKC was also evaluated. (**D**) SMF cells were treated with either DMSO or STU for 24 hours at the indicated concentration. Immunoblotting for p27 was performed using anti-p27 antibody. (**E**) SMF cells plated on coverslips were treated with either DMSO or 1 nM STU for 24 hours before fixation and subsequent confocal imaging. (**F**) Percent of cells containing nuclear p27 in SMF cells treated with DMSO or STU.

Given that our structural model of STU in the kinase pocket of HUNK suggests a reasonable interaction, we wanted to experimentally evaluate whether STU treatment would lead to a similar cell biological outcome as HUNK inhibition. STU is a well known apoptosis inducing agent [13–16] and HUNK has been described to promote cell survival [6, 9–11]. Therefore, we next wanted to determine the effect of STU on cell death in HUNK-deficient cells. We reasoned that removing HUNK as the STU target would diminish the effect of STU on cell death, if HUNK is a major target of STU. To test this concept, we took SMF cells expressing either a control shRNA or shRNA targeting mouse HUNK (*Hunk*) to reduce *Hunk* mRNA levels (Figure 4A), and treated the cells with 5 nM STU to measure caspase-3 activity as a surrogate for caspase-dependent apoptosis. We reasoned that the SMF cell line was a practical model to evaluate these effects since prior work had shown that these cells are dependent on HUNK for survival [6]. We also used growth factor deprivation as a secondary means of inducing cell death. Interestingly, while we saw that *Hunk* knockdown cells were sensitive to loss of growth factors and exhibited significant caspase-3 activity, STU treated *Hunk* knockdown cells did not significantly induce caspase-3 activity compared to control cells (Figure 4B). We concluded that the decreased HUNK expression in these cells reduced levels of the STU target, thereby lessening the cytotoxic effect of STU.

Because one of the major targets of STU is protein kinase C (PKC) [17], we also wanted to determine the effect of STU on PKC activity in the SMF cells. To evaluate PKC activity, we treated SMF cells with either 1 nM or 5 nM STU, and probed for the phosphorylation of PKC substrates using a phospho-specific PKC substrate antibody as well as a phospho-specific antibody for PKC itself. We found that levels of PKC phosphorylation as well as phosphorylation of PKC substrates were unchanged due to STU treatment, compared to cells treated with DMSO (Figure 4C), suggesting that the doses of STU that we used were not sufficient to inhibit PKC activity.

Prior studies showed that HUNK downregulates expression of the tumor suppressor p27 by preventing nuclear accumulation of p27 [6, 7]. Therefore, to further evaluate the potential effect of STU on HUNK function, we next assessed p27 expression and localization in SMF cells after STU treatment. To determine if applying STU to SMF cells resulted in stabilized p27 expression, we treated SMF cells with 1 nM or 5 nM STU and examined p27 protein expression levels by western blotting. We found that STU treatment, at either dose, stabilized p27 expression in the SMF cells (Figure 4D) similar to what has been observed in SMF cells expressing *Hunk* shRNA [6]. Corresponding with the increase in p27 stability, STU treatment also resulted in an increase in p27 nuclear localization compared to treatment with DMSO (vehicle) as previously described [6]; immunofluorescence (Figure 4E) and quantitation (Figure 4F). Collectively, these results indicate that STU exhibits an activity toward cells that is the same as what is observed with studies that inhibit HUNK by other means (e.g. shRNA knockdown or K91M expression).

### Staurosporine synergizes with lapatinib in HER2 inhibitor resistant breast cancer cells

**Figure 5:**
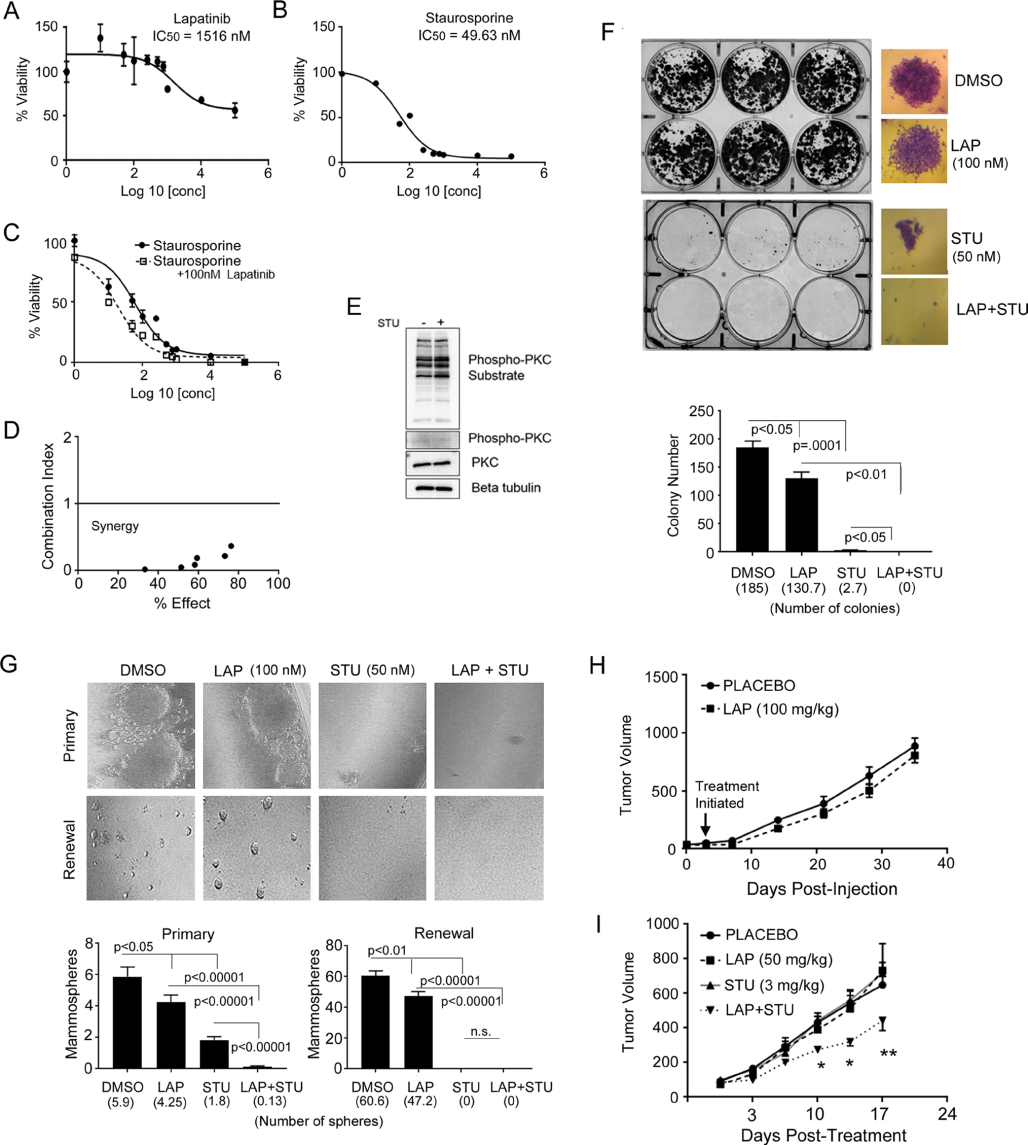
STU synergizes with lapatinib (**A**) IC_50_ value was generated by evaluating viability of JIMT-1 cells treated with increasing doses of lapatinib to show these cells are inherently resistant to lapatinib. (**B**) IC_50_ value was generated by evaluating viability of JIMT-1 cells treated with increasing doses of STU. (**C**) Dose response curves and IC_50_ value were generated by evaluating viability of JIMT-1 cells treated with increasing doses of STU in combination with 100 nM lapatinib. (**D**) Combination index analysis indicates that STU is synergistic with 100 nM lapatinib at all concentrations evaluated, with a score below “1” indicating synergy. (**E**) JIMT-1 cells were treated with either DMSO or 25 nM STU for 24 hours. Immunoblotting for PKC activity was performed using anti-phospho-PKC, anti-phospho-PKC substrate, and anti-PKC antibodies. (**F**) To assess colony formation, JIMT-1 cells were initially plated in normal growth medium, followed by next day replacement of media containing drug (DMSO, 100 nM lapatinib, 25 nM STU, or combination 100 nM lapatinib plus 25 nM STU). Cells were treated with drugs for 3 days, followed by replacement with normal growth media for the next 21 days. At day 21, colonies were stained with crystal violet and quantified. (**G**) To assess mammosphere formation and renewal, JIMT-1 cells were plated in media containing either DMSO, 25 nM STU, 100 nM lapatinib, or combination 25 nM STU plus 100 nM lapatinib. Cells were maintained in drug for 14 days, then quantified and imaged. To assess renewal, primary mammospheres were trypsinized and re-plated in drug containing media at equal numbers. Mammosphere formation was quantified 14 days after primary mammospheres were plated for renewal. (**H**) JIMT-1 cells were injected into the mammary fat pad of immunocompromised mice to assess mammary tumor growth. Mice received placebo or 100 mg/kg lapatinib twice a week for the duration of the experiment. (**I**) To assess combined delivery of STU and lapatinib, JIMT-1 cells were injected into the mammary fat pad of immunocompromised mice to assess mammary tumor growth. Mice were randomized into either placebo, STU (3 mg/kg), lapatinib (50 mg/kg), or combination STU plus lapatinib treatment groups. When tumors reached a size of ∼65 mm^3^, treatments were initiated and administered twice weekly. Tumors were analyzed by student’s *T*-test at individual time points. ^*^p < 0.001; ^**^p = 0.02.

Previous studies indicate that *HUNK* knockdown in trastuzumab/lapatinib-resistant, HER2+ JIMT-1 cells shows decreased orthotopic mammary tumor growth [9, 10]. Because our findings demonstrate that STU inhibits HUNK kinase activity, we wanted to evaluate the response of JIMT-1 cells to STU treatment alone and in combination with HER2 inhibition. First, we evaluated the level of sensitivity of JIMT-1 cells to lapatinib. Consistent with prior reports, JIMT-1 cells were relatively resistant to lapatinib *in vitro* (Figure 5A). We calculated an IC_50_ for lapatinib treated JIMT-1 cells to be in the µM range (∼1.5 µM). Because prior reports indicate that inhibiting HUNK is effective in reducing cell viability of JIMT-1 cells [9, 10], we reasoned that JIMT-1 cells would show greater sensitivity to STU than lapatinib. When we performed dose response analysis of STU treatment on JIMT-1 cells, we found the calculated IC_50_ of STU (∼50 nM) to be ∼30 times lower than that of lapatinib (Figure 5B).

We then sought to investigate whether STU can synergize with lapatinib in drug resistant HER2+ breast cancer cells. Therefore, we evaluated JIMT-1 cell viability after co-treatment with STU and lapatinib, where we varied the dose of STU but kept the lapatinib concentration constant. Furthermore, the dose of lapatinib (100 nM) used was >10 times lower than the observed IC_50_ for lapatinib (∼1.5 µM) in the JIMT-1 cells, shown in Figure 5A. We found that combining STU and lapatinib resulted in a greater suppression of cell viability compared to STU treatment alone (Figure 5C). Combination index analysis demonstrated synergy (denoted as a score below 1) between STU and lapatinib at all doses of STU (Figure 5D). To ensure that the effect of STU on viability in the JIMT-1 cells was not due to inhibition of PKC, we treated these cells with STU and examined phosphorylation of PKC substrates and PKC. We found that, similar to our results in the SMF cell line, PKC and PKC substrate phosphorylation were unchanged with STU treatment (Figure 5E).

Because STU and lapatinib showed synergistic effects on viability, we wanted to determine if STU and lapatinib showed similarly potent effects on other physiological outcomes. Therefore, we next performed a colony formation assay using the JIMT-1 cells treated with either DMSO, STU, lapatinib, or the combination of STU and lapatinib using doses below the IC_50_ of each inhibitor. While 25 nM STU showed modest effects on suppressing colony formation, we found that the combination treatment of 25 nM STU with 100 nM lapatinib significantly reduced colony formation, similar to our viability results (Figure 5F). Next, we evaluated mammosphere formation, and found that combination treatment of STU and lapatinib showed stronger effects on suppressing primary mammosphere formation than either agent alone (Figure 5G). Following analysis of the primary mammospheres, we also looked at mammosphere renewal and found that STU treatment, either alone or in combination with lapatinib, completely prevented mammosphere self-renewal (Figure 5G).

To determine if the effects of STU and lapatinib that we observed *in vitro* was indicative of an effect *in vivo*, we orthotopically transplanted JIMT-1 tumor cells into the mammary glands of immunocompromised mice to evaluate tumor growth with individual and combination drug treatments. To first evaluate the level of resistance that tumors would exhibit to lapatinib in our hands, we performed a control experiment where we orthotopically transplanted JIMT-1 tumor cells into mammary glands and treated animals with either a placebo or a 100 mg/kg dose of lapatinib. Tumors exhibited a strong resistance to lapatinib under this paradigm showing no difference in tumor growth in comparison to tumors from placebo treated animals (Figure 5H). We next performed an experiment to evaluate single agent lapatinib or STU treatment with the combination of the two drugs. For this analysis, we used a 50 mg/kg dose of lapatinib, which was lower than the 100 mg/kg dose of lapatinib that we showed tumors were resistance to, in Figure 5H. Once tumors formed, mice were randomly assigned to one of four groups: placebo, lapatinib, STU, or STU in combination with lapatinib and evaluated for tumor formation. Consistent with Figure 5H, we saw no effect of 50 mg/kg lapatinib treatment on tumor growth. We also found that STU by itself showed no effects on tumor growth, likely due to the fact that we used a low dose of STU (3 mg/kg). However, we saw that the combination of 3 mg/kg STU and 50 mg/kg lapatinib impaired tumor growth in a statistically significant manner (Figure 5I). Further analysis conveyed that the combined treatment group grew at a rate that was significantly slower than the single treatment groups (*p* ≤ 0.001 in all cases for combined slope vs. single treatment slopes; Wald test). The tumors in the lapatinib and STU combined treatment group grew with an average change per day in volume of 21 mm^3^/day compared to single treatment groups at 33.5 mm^3^/day for placebo, 35.9 mm^3^/day for lapatinib, and 36.9 mm^3^/day for STU. Collectively, these results suggest that STU and lapatinib show a potent anti-tumor effect when applied in combination against HER2 inhibitor resistant breast cancer cells.

## DISCUSSION

Despite the diverse roles kinases play within cells, the kinase domain is structurally conserved between many proteins. With approximate 500 human kinases, ATP-mimetic analogs are likely to have off-target binding to the ATP binding pockets of other homologues creating a major challenge in the design of kinase inhibitors [18, 19]. However, the demonstration of successful design of high-affinity selective ATP-mimetic drugs has allowed kinases to be the mainstay as attractive targets, particularly in cancer therapeutics. Structure-based computational aided drug design (CADD) can be used in the design of potent and selective inhibitors of kinases but relies on high quality 3D structure from either crystal structure, NMR or homology models. While no experimental structures are available for HUNK, a wealth of information pertaining to kinase domain structures have been deposited to the protein data bank (PDB), with ∼9250 protein kinase entries at the time of this study.

A crystal structure can be considered a snapshot of a dynamic and highly flexible system and can change considerably depending on conditions and ligands present when crystallized. These considerations need to be taken into account when studying homologous proteins, such as protein kinases. Consequently, the use of ligand-steered homology modeling becomes critical. It is therefore important to not only select templates based on sequence identity and similarity but also consider the ligands co-resolved within the template structure. While not absolute, our model of HUNK and STU takes into account these factors and ultimately, serves to provide a platform for modeling interactions between chemical compounds and the HUNK enzymatic domain. Our modeling shows proof of principle that we have developed high quality models of HUNK that can be used in future studies to identify and guide the drug discovery process.

STU is a broad-spectrum protein kinase inhibitor that was first isolated from the bacterium *Streptomyces staurosporeus* [17]. STU is classified as a competitive kinase inhibitor that competes with ATP for interaction in the binding pocket of its target kinases. It is most commonly known as a PKC inhibitor, but has been reported to bind multiple protein kinases with high affinity [20, 21]. STU has been investigated for its potential anti-tumor activity in several cancer cell lines, including cervical, colon, oral, and breast [15, 22–24]. This is due to STU’s ability to induce apoptosis in multiple cell lines *in vitro* [25]. However, preclinical studies testing STU as an anti-cancer therapeutic have been largely unsuccessful due to its high potency and poor target specificity. Past clinical and preclinical studies using STU analogs, such as UCN-01, lestaurtinib, and midostaurin, show that these compounds have reduced toxicity compared to STU. While these analogs have been tested in a number of clinical trials for various cancer subtypes, including leukemia, lymphoma, melanoma, as well as solid tumors, UCN-01 and lestaurtinib have not done well due to their off-target effects. However, midostaurin is still actively being tested in clinical trials [26]. Our findings allude to the need for identifying specific types or subtypes of cancers that can best benefit from agents that have broad actions due to multiple targets.

Preclinical studies have indicated HUNK’s potential as a novel therapeutic target in HER2+ breast cancer [6, 7, 9, 10]. To date, HUNK inhibition has been accomplished either through the use of shRNA to decrease HUNK expression, or through the use of kinase-inactive HUNK mutants, such as K91M HUNK [6, 8, 11]. We show for the first time that STU is a novel inhibitor of HUNK kinase activity, providing a pharmacological inhibitor that can now be used experimentally to inhibit HUNK enzymatic activity. We further demonstrate that the pharmacological activity of STU leads to a reduction in viability of HER2 inhibitor resistant breast cancer cells. Since we show that STU inhibits HUNK kinase activity by biochemical assay, as a whole, these findings support previous studies that indicate HUNK inhibition is likely beneficial in the treatment of HER2+ resistant breast cancer.

Despite the clinical availability of HER2 inhibitors such as trastuzumab and lapatinib, their efficacy is often short-lived due to high rates of acquired resistance [27]. Identifying ways to overcome resistance remains a major barrier to the treatment of HER2+ breast cancers. Our collective results, past and present, indicate that HUNK inhibition is effective in HER2+ breast cancer models [9, 10] and application of STU acts synergistically with the HER2 inhibitor lapatinib in JIMT-1 cells, a cell line inherently resistant to lapatinib. Our findings are supported by *in vivo* experiments that show the combination of STU and lapatinib suppresses colony formation and mammosphere formation in the JIMT-1 cell line. Most strikingly, STU treatment either alone or in combination with lapatinib, completely abolished mammosphere renewal. Because breast cancer stem cells are implicated in HER2 inhibitor resistance, identifying targets like HUNK that potentially regulate self-renewal capability may aid in preventing acquired resistance. Our results are further supported by our findings that giving a low dose combination of STU and lapatinib to mice with tumors generated by orthotopic transplantation of JIMT-1 cells, results in a suppression of tumor growth. Taken together, these findings suggests that HUNK inhibition may sensitize resistant cells to HER2 inhibition *in vivo*.

In summary, our findings indicate that the kinase inhibitor, STU, inhibits HUNK kinase activity. We show that STU suppresses cell viability in HER2+/neu mammary and breast cancer cell lines. Furthermore, we demonstrate that STU synergizes with lapatinib in HER2 inhibitor resistant breast cancer cells *in vitro* and *in vivo*. Collectively, our studies support the therapeutic potential of targeting HUNK in HER2+ breast cancer cells and indicate that targeting HUNK in combination with HER2 inhibition may be a possible option for HER2+ resistant breast cancers.

## MATERIALS AND METHODS

### Cell culture

All cell lines were kept at 37° C and 5% CO_2_. 293T cells and JIMT-1 cells were grown in DMEM (Corning) supplemented with 10% fetal bovine serum (FBS, Gibco). SMF cells were grown in DMEM (Corning) supplemented with 10% FBS and 5 mg/ml insulin (Gemini Bio-Products). All media contained 2 mM glutamine (Corning) and penicillin/streptomycin (Corning), unless stated otherwise. Lapatinib was purchased from Santa Cruz Biotech. Axitinib, Dovitinib, KW-2449, staurosporine, UCN-01, midostaurin, lestaurtinib, PHA-665752, SU-14913, and sunitinib were purchased from Selleck Chemicals.

### Viability assay

Equal numbers of cells were plated and treated the following day with increasing doses of either staurosporine, UCN-01, or lestaurtinib in media containing 1% FBS. After 48 hours of drug treatments, cells were fixed in 4% paraformaldehyde and stained with crystal violet for 1 hour. Cells were then washed in deionized water and left to dry. Methanol was then used to extract crystal violet stain. Absorbance 540 was read using the Molecular Devices FilterMax F5 microplate reader.

### Caspase-3 activity assay

Equal numbers of cells (20,000 cells/well) were plated on 96-well dishes and treated the following day with STU for 24 hours prior to analysis by Caspase-3 activity assay (Sigma) as previously described [10].

### Immunoblotting

All cells were lysed in buffer containing 50 mM Tris-HCl, pH 7.5, 150 mM sodium chloride, 1 mM EDTA, 1% Triton X-100 with HALT protease and phosphatase inhibitor cocktail (Thermo Scientific). Primary antibodies used for western blotting include: anti-phospho-MBP (EMD Millipore, 05-429), anti-MBP (LifeSpan BioSciences, LS-C312288/59980), anti-flag-M2 (Sigma-Aldrich, #F1804), anti-HUNK (Invitrogen PA5-28765), anti-p27 (Santa Cruz, sc-528), anti-phospho-PKC-substrates (Cell Signaling, #2261), anti-phospho-PKC (Santa Cruz, sc-271920), anti-PKC (Santa Cruz, sc-17769), and anti-β-tubulin (Santa Cruz, sc-55529). Imaging was performed on the Protein Simple FluorChem-R imaging system.

### Kinase assay

Flag-Hunk was expressed in 293T cells and immunoprecipitated (IP) with anti-flag M2 magnetic beads (Sigma, M8823). For kinase reactions, the IP kinase was first incubated in kinase buffer (20 mM HEPES 2 mM MgCl pH 7.3), 500 uM ATP, 20 mM HEPES 2 mM MgCl, and inhibitors at a 1 µM or 5 µM final concentration as indicated, prior to addition of substrate; MBP (EMD Millipore). All kinase reactions were incubated at 30° C for 15 minutes.

### Immunofluorescence

For p27 nuclear analysis, equal numbers of SMF MMTV-neu cells were plated on gelatin-coated coverslips (Corning, No. 1.5). Prior to imaging, cells were treated with either DMSO or 1 nM staurosporine for 24 hours. Cells were fixed with 4% paraformaldehyde, followed by permeabilization with 0.1% Triton X-100 in PBS. Anti-p27 (Santa Cruz, sc-528) and goat-anti-rabbit Alexa Fluor 594 (Life Technologies) antibodies were used for staining. Images were captured using a Leica DMi8 Confocal Microscope.

### Mammosphere assay

500 cells per well were plated into cell surface repellent 96-well culture plates in DMEM/HamsF12 media (Gibco) supplemented with B27 (Gibco), EGF (Sigma), basic FGF (Invitrogen), and heparin (Stemcell Technologies). On the day of plating, mammospheres were treated with either DMSO, 25 nM staurosporine, 100 nM lapatinib, or a combination of staurosporine and lapatinib. Cells were maintained in culture for 14 days before quantitating mammospheres. For the mammosphere renewal assay, mammospheres were trypsinized and re-plated at 500 cells per well in media containing the same drug concentrations used for the primary assay. Mammosphere renewal was quantified 14 days after re-plating.

### Colony formation assay

2000 cells per well were plated into 6-well plates. Cells were initially plated in normal growth media for 24 hours, followed by media replacement containing DMSO, 25 nM staurosporine, 100 nM lapatinib, or a combination of the two. Cells were incubated in media containing drug for 3 days, followed by replacement with normal growth media. Growth media was refreshed every 2 days. At day 21, colonies were stained with crystal violet, and imaged using the Protein Simple FluorChem-R imaging system. Colony count and area were quantified using the AlphaView Software.

### RNA isolation and quantitative RealTime PCR

RNA was isolated using the GeneJet RNA isolation kit (Thermo Scientific). Reverse transcription was performed using the Maxima First Strand cDNA Synthesis Kit for RT-PCR (Thermo Scientific). RealTime PCR using PrimePCR mouse *Hunk* (Bio-Rad) was performed using the Bio-Rad myIQ. *Hunk* mRNA levels were normalized to *Gapdh* levels. Primers for *Gapdh* are: Forward-GCACAGTCAAGGCCGAGAAT, Reverse-GCCTTCTCCATGGTGGTGAA

### Animal care

All animal experiments were approved by the Medical University of South Carolina IACUC. All animals were housed and cared for in the AAALAC accredited Animal Research Center at Medical University of South Carolina and routinely monitored by lab and veterinary staff. Animals were housed in a BSL2 facility for immunocompromised animals in individually ventilated racks with sterile water and food. Animals were euthanized by isofluorane overdose in accordance with the Guide for the Care and Use of Laboratory Animals. Protocols were in place for early and humane endpoints in the event that an experimental animal displayed signs of illness, such as poor body condition, lethargy, piloerection, and lack of grooming behavior, prior to the experimental endpoint. To determine if and when animals should be euthanized, tumor measurements and health monitoring of experimental animals was performed regularly by lab and veterinary staff.

### *In vivo* tumorigenesis

For each cohort, 5 × 10^6^ JIMT-1 cells were injected into a single abdominal mammary fat pad of a female immunocompromised mice, Nu/J-Foxn1 ^nu/nu^ (Jackson Labs). Tumor size was measured manually using calipers. When tumors reached a volume of ∼65 mm^3^, mice were randomized into one of four treatment groups: placebo, STU, lapatinib, or combined STU and lapatinib. Drugs were resuspended in a solution containing 0.5% hydroxypropylmethylcellulose, 0.1% Tween-20, 50% DMSO, which was the vehicle used for the placebo group. For the STU single and combination treatment groups, mice received 3 mg/kg STU via oral gavage twice a week. For the lapatinib single and combination treatment groups, mice received 50 mg/kg lapatinib via oral gavage twice a week. Tumor measurements were taken twice weekly on the days of treatment.

### Statistical analysis

Combination index analysis was performed with the CompuSyn software using the Chou-Talalay method as previously described [28]. IC_50_ was calculated using GraphPad Prism software. Linear mixed-effects regression was used to model tumor growth for the single agent vs. combination treatment, with a random component to account for correlation of repeated measures over time within animals. General linear hypothesis tests were used to test for differences in slopes between group pairs using linear combinations of the resultant model coefficients. For all other analyses, student’s *T*-Test was used; error bars in plots represent the Standard Error of the Mean.

### Homology modeling

The *H. sapiens* HUNK sequence was retrieved from Uniprot (P57058) and used in a protein-protein blast against the Protein Data Bank (PDB). The top scoring PDB structures were retrieved. In addition all protein kinase structure resolved with staurosporine was retrieved from the Protein Data Bank (PDB). A protein sequence alignment was performed between HUNK and the retrieved protein kinases using MUSCLE [29]. Manual edits were performed using Discovery Studio (Dassault Systèmes BIOVIA, Discovery Studio, Release 2017, San Diego: Dassault Systèmes, 2018). Homology models bound with staurosporine were generated from two template structures (PDB entries: 1NVR and 1WVY) using modeler 9v19 [30]. Amino acids are numbered with the initiating methionine set to 1. The models were subjected to quality analysis using the PDBsum generator (http://www.ebi.ac.uk/pdbsum) [31]. The models were prepared for analysis using the protein preparation wizard in which protonation states were assigned followed by an energy minimization to relieve unfavorable constraints (Schrödinger Release 2017-3 Schrödinger, LLC, New York, NY, 2017).

### Molecular dynamics

A 0.5 ns molecular dynamics run was performed at 100K to equilibrate the HUNK model structure containing staurosporine to further relieve unfavorable constraints. The complex was prepared using the Desmond (Schrödinger Release 2017-3 Schrödinger, LLC, New York, NY, 2017). Ligand and protein were treated with the OPLS3 force field and solvated using the SPC water model. The overall system was neutralized at pH 7.0 using a 0.15 M ion concentration of NaCl. The final structure from this simulations was capture subjected to minimization and used in further docking studies.

### Molecular docking

All compounds were treated using ligprep (Schrödinger Release 2017-3 Schrödinger, LLC, New York, NY, USA, 2017). Protonation states were predicted using epic with a pH range of 7.0 ± 2.0. GRIDs were generated using staurosporine as the center of the binding pocket with the inner box set to 10 Å and the outer box to 30 Å. All docking was performed using Glide in combination with the extra-precision (XP) scoring function [32] and the OPLS3 force field (Schrödinger Release 2017-3 Schrödinger, LLC, New York, NY, 2017) [33]. The best scoring binding poses were kept and subjected to post docking minimization using the OPLS3 force field with a rejection threshold of 0.5 kcal/mol [33].
